# Comparison of activation patterns between masking and inattention tasks: a coordinate-based meta-analysis of implicit emotional face processing

**DOI:** 10.3389/fnhum.2013.00459

**Published:** 2013-08-26

**Authors:** Huqing Shi, Xiang Wang, Shuqiao Yao

**Affiliations:** ^1^Medical Psychological Institute, Second Xiangya Hospital, Central South UniversityChangsha, China; ^2^Key Laboratory of Psychiatry and Mental Health of Hunan Province, Second Xiangya Hospital, Central South UniversityChangsha, China

**Keywords:** emotional faces, fMRI, implicit processing, masking, inattention

## Abstract

Neuroimaging studies of implicit emotional processing are important for understanding the neural mechanisms and its social and evolutionary significance. Two major experimental tasks are used to explore the mechanisms of implicit emotional processing: masking tasks and inattention tasks, both using emotional faces as stimuli. However, it is unclear whether they have identical or distinct neural substrates since few studies have compared the two tasks. The purpose of the present study was to explore the mechanisms of implicit processing of emotional faces, and compare the activation patterns between different tasks. Through a literature search, 41 studies exploring implicit processing of emotional faces were collected. A total of 830 healthy subjects and 513 foci were obtained. Separate activation likelihood estimation (ALE) meta-analyses were conducted for the entire group of studies and for different tasks for comparison purposes. The results showed that there were differences, as well as overlap, in activation patterns between masking and inattention tasks. Bilateral amygdala, middle occipital gyrus and fusiform gyrus were activated across both tasks. While masking tasks were more associated with inferior temporal gyrus, parahippocampal gyrus and amygdala, inattention tasks were more associated with right fusiform gyrus. The differences in activation patterns between masking and inattention tasks may be indicative of separate mechanisms underlying early and late stages of implicit emotional face processing.

## Introduction

Human facial expressions are powerful non-verbal emotional cues which elicit direct and automatic responses. Many studies therefore, have focused on the neural substrate of emotional face processing. Activation of the occipital regions, middle and superior temporal gyrus, limbic regions, as well as ventral and medial prefrontal regions has been reported during facial expression processing (Haxby et al., 2000; Vuilleumier and Pourtois, 2007). Implicit processing has become a popular research interest, since it occurs in the early stages of attention and cognition, may reflect more social and evolutionary significance of emotion compared to explicit processing. Neuroimaging studies have revealed the involvement of many brain areas in implicit emotional processing, including the amygdala, thalamus, insula, the fusiform gyrus, anterior cingulate and the inferior frontal gyrus (Critchley et al., 2000a). Occipital regions such as the left lingual gyrus, right fusiform gyrus, left postcentral gyrus and right insula have also been shown to be involved (Fusar-Poli et al., 2009a). However, the above studies have employed a variety of experimental tasks and designs leading to confounding variables and disparate results.

Previous research has explored the underlying mechanism of implicit emotional processing through a variety of methods (Morris et al., 1999; Killgore and Yurgelun-Todd, 2004). Two major tasks have been widely used to access the implicit processing, masking task and inattention task. The most popular masking task is backward masking in which a target emotional stimulus is presented for a very short period (<40 ms) followed immediately by a neutral stimulus such as a neutral face (as a mask) (Esteves and Ohman, 1993). Some researchers refer to this type of processing as subliminal processing, where the stimuli are attended to by the brain, but are too short or weak to be consciously perceived (Dehaene et al., 2006; Pessiglione et al., 2007, 2008). The most common contrasts used to measure neural activities for masked emotional stimuli were neutral stimuli, sometimes baseline conditions (such as fixation cross) (Pine et al., 2001; Killgore and Yurgelun-Todd, 2004; Phillips et al., 2004). The strength of masked emotional stimuli could activate subcortical regions such as the amygdala (LeDoux, 2003; Phillips et al., 2004). A rapid neural pathway from the amygdala to the early visual cortex is regarded to be involved in the subliminal emotional processing (LeDoux, 1996).

On the other hand, the most representative method of inattention task is dual-task paradigm. In this design the subject's attention is distracted by a non-emotional task such as determining the gender of the face while an emotional face is presented, or determining whether the houses (non-emotional objects) are identical while emotional faces are presented at the same time (Vuilleumier et al., 2001; Anderson et al., 2003). In this case, the stimuli could be consciously perceived, but stay outside of attentional focus. The attentional process is involved, but works against emotional processing by allocating attentional resources to non-emotional tasks. The most common contrasts used to measure neural activities for unattended emotional stimuli were also neutral stimuli, sometimes baseline conditions (such as fixation cross) (Lobaugh et al., 2006; Harrison et al., 2009). Some studies used contrasts such as unattended vs. attended stimuli were not investigated in this study (Williams et al., 2005; Ewbank et al., 2009). The unattended emotional stimuli could activate cortical visual cortex such as primary visual cortex (V1) (Tamietto and de Gelder, 2010). According to Kouider and Dehaene (2007), conscious perception is prevented either by insufficient stimuli strength or insufficient top–down attention, which corresponding to masking tasks and inattention tasks, respectively. That is, masking tasks reduce bottom-up input for emotional stimuli, and inattention tasks reduce top–down attention for emotional stimuli. This raises the question of whether these two tasks would lead to divergence in activation patterns of implicit emotional face processing.

Also, there has been a debate on perceptual load and emotional processing. The load theory of selective attention suggests that high perceptual load tasks have suppressive effects on task-irrelevant stimuli when the attentional resources are limited (Lavie, 2005). Some studies show reduced activities when the non-emotional tasks are demanding (Pessoa et al., 2002, 2005; Pessoa, 2005; Silvert et al., 2007). However, it remains unclear whether the implicit emotional processing would interact with different levels of perceptual load.

Based on the above question, the aim of the current study was to examine effects of experimental tasks on brain activation of emotional stimuli through an activation likelihood estimation (ALE) meta-analysis of fMRI studies (Turkeltaub et al., 2002). This method has the advantage of increasing sample size and extracting specific activated brain areas from different studies to understand a full representation of the activation patterns. Masking tasks and inattention tasks both reveal underlying mechanisms of implicit emotional processing but place emphasis on different stages. The present study analyzed these two processes together and separately. Sub-analyses for different perceptual load were also carried out to examine possible effects. Based on previous research, we hypothesized that the brain activation patterns would differ between tasks. Masking tasks would be more involved with sub-cortical areas such as amygdala; inattention tasks would be more involved with cortical areas such as prefrontal cortex. Also, there would be overlapping areas showing consistent activation patterns for implicit emotional face processing.

## Methods

### Literature search and inclusion criteria

An online search of journal articles from January 1993 to June 2013 via PubMed, Web of Science was conducted by using key words “emotional face,” “fMRI,” “implicit,” “covert,” “masking,” “inattention,” “unattended,” “distract,” “dual-task,” etc. Each being fully examined, articles were selected based on the following inclusion criteria: (1) fMRI studies published in peer reviewed journals with healthy subjects; (2) used emotional faces as experimental stimuli; (3) used experimental paradigms implied implicit emotional processing (such as masking task, distraction task, dual-task, etc.); (4) used image subtraction methodology, results reported in emotional vs. neutral contrasts; (5) results reported as normalized spatial coordinates, either in Montreal Neurological Institute (MNI) or Talairach space. Literature were excluded for one or more reasons below: (1) used non-fMRI techniques, such as positron emission tomography (PET) or event-related potential (ERP) studies; (2) used other types of emotional stimuli (such as emotional pictures or scenes), or used faces in a non-emotional way (such as familiar faces); (3) experimental paradigms focused on psychological processes which implied only explicit processing, such as emotional faces recognition or recollection; (4) no specific emotional vs. neutral contrasts available, such as emotional vs. baseline contrasts, correlational studies or functional connectivity studies; (5) no data available in standard spatial coordinates; (6) results from case studies or reviews. Although studies with psychiatric patients were not excluded, only the results from healthy controls were included in the meta-analyses. Both negative and positive emotional stimuli were included in the meta-analyses, but separate sub-analyses were carried out to examine possible influence of emotional valence. Similarly, separate sub-analyses were carried out for event-related and block design studies, as well as sub-analyses for studies with high and low perceptual load tasks.

### Basic description of included articles

Two reviewers independently screened the literature using the above criteria. Reference lists of the selected literature were also checked for potential inclusion. A total of 41 studies with 830 subjects, 513 foci were included in the ALE meta-analysis. Table 1 presents all the literature in this study, details are showed for: (1) demographic characteristics; (2) experimental stimuli, paradigm, and design; (3) field strength; (4) fMRI analysis methods, contrasts, and significance threshold.

**Table 1:** **Included studies and descriptive variables**.

**Study (first author, year)**	**Number of subjects**	**Stimuli**	**Presentation time (ms)**	**Experimental paradigm**	**Field strength**	**fMRI methods (design, analysis)**	**Contrasts**	**Threshold**	**Number of foci**
Anderson et al., 2003	3m, 9f	Fearful, disgusted faces	750	Dual-task (gender-decision)	3T	Event-related, ROI	Emotional vs. neutral	*p* < 0.01	2
Anderson et al., 2007	12 m	Angry, disgusted faces	3000	Gender-decision	1.5T	Block, whole-brain and ROI	Emotional vs. neutral	WB: uncorrected *p* < 0.001, >10 voxels; ROI: *p* < 0.05, SVC	29
Anderson et al., 2011	2m, 10f	Happy, sad, fearful faces	3000	Gender-decision	1.5T	Block, ROI	Emotional vs. neutral	Uncorrected *p* < 0.001, >5 voxels	9
Attar et al., 2010	9m, 11f	Fearful, happy faces	2000	Dual task (high attentional load)	3T	Event-related, whole-brain and ROI	Emotional vs. neutral	WB: FWE *p* < 0.05; ROI: *p* < 0.05, SVC	4
Batut et al., 2006	6m, 9f	Happy, sad, fearful faces	3000	Gender-decision	2T	Block, whole-brain	Emotional vs. neutral	Uncorrected *p* < 0.001, >10 voxels	21
Bentley et al., 2003	8m, 7f	Fearful faces	250	Dual task (high attentional load)	2T	Block, whole-brain and ROI	Emotional vs. neutral	Uncorrected *p* < 0.01	2
Bishop et al., 2004	7m, 20f	Fearful faces	250	Dual task (high attentional load)	3T	Block, ROI	Emotional vs. neutral	Corrected *p* < 0.05	1
Bryant et al., 2008	7m, 8f	Fearful faces	16.7	Masking task	1.5T	Block, ROI	Emotional vs. neutral	*p* < 0.05, >3 voxel, SVC	3
Critchley et al., 2000a	9m	Happy, angry faces	3000	Gender-decision	1.5T	Block, whole-brain	Emotional vs. neutral, emotional vs. baseline	*p* < 0.01	18
Dannlowski et al., 2007	12m, 11f	Sad, angry, happy faces	33	Masking task	3T	Block, ROI	Emotional vs. neutral	Corrected *p* < 0.05	8
Del-Ben et al., 2005	12m	Angry, disgusted, fearful faces	3000	Gender-decision	1.5T	Block, whole-brain and ROI	Emotional vs. neutral	Uncorrected *p* < 0.001, >10 voxels	15
Duan et al., 2010	5m, 13f	Surprised, happy faces	33	Masking task	3T	Block, whole-brain	Emotional vs. neutral	Uncorrected *p* < 0.001, >5 voxels	41
Habel et al., 2007	15m, 14f	Happy, sad, angry, fearful, disgusted faces	5000	Age-judgment	3T	Event-related, whole-brain and ROI	Emotional vs. neutral	FWE *p* < 0.05	9
Hall et al., 2010	12m	Anxious faces	33	Masking task	3T	Event-related, ROI	Emotional vs. neutral	FDR *p* < 0.05	2
Harrison et al., 2009	16m	Happy, sad, angry faces	500	Age-judgment	1.5T	Event-related, whole-brain and ROI	Emotional vs. baseline emotional vs. neutral	FWE *p* < 0.05	11
Jehna et al., 2011	9m, 21f	Angry, fearful, disgusted faces	3000	Gender-decision	3T	Block, whole-brain	Emotional vs. neutral	Corrected *p* < 0.05	4
Killgore and Yurgelun-Todd, 2007a	12f	Happy, sad faces	20	Masking task	1.5T	Block, whole-brain	Emotional vs. neutral	Uncorrected *p* < 0.001, >20 voxels	45
Killgore and Yurgelun-Todd, 2007b	2m, 8f	Happy, sad faces	20	Masking task	1.5T	Block, whole-brain and ROI	Emotional vs. neutral	WB: *p* < 0.005, >20 voxels; ROI: *p* < 0.05, >10 voxels	22
Liddell et al., 2005	11m, 11f	Fearful faces	16.7	Masking task	1.5T	Block, whole-brain and ROI	Emotional vs. neutral	WB: uncorrected *p* < 0.001, >3 voxels; ROI: *p* < 0.05, >3 voxels, SVC	19
Lobaugh et al., 2006	6m, 3f	Digusted, fearful, angry, sad, surprised, happy faces	500	Gender-decision	1.5T	Block and event-related, whole-brain	Emotional vs. baseline emotional vs. neutral	*p* < 0.001	6
Monk et al., 2008	6m, 6f	Angry faces	17	Masking task	3T	Event-related, whole-brain and ROI	Emotional vs. neutral	Uncorrected *p* < 0.001	3
Nomura et al., 2004	15f	Angry faces	35	Masking task	3T	Event-related, whole-brain	Emotional vs. neutral, emotional vs. baseline	Uncorrected *p* < 0.05, >7 voxels	8
Norbury et al., 2007	6m, 6f	Happy faces	17	Masking task	1.5T	Block, whole-brain	Emotional vs. neutral	Corrected *p* < 0.05	1
Palm et al., 2011	16f	Fearful, angry, happy faces	3250	Gender-decision	1.5T	Block, whole-brain	Emotional vs. neutral	Uncorrected *p* < 0.001, >10 voxels	12
Pessoa, 2005	19m, 18f	Fearful faces	33	Masking task	1.5T	Event-related, ROI	Emotional vs. neutral	*p* < 0.05	3
Posner et al., 2011	13m, 2f	Fearful faces	30	Masking task	3T	Block, whole-brain	Emotional vs. neutral	*p* < 0.001, AlphaSim corrected	7
Rauch et al., 2007	10m, 10f	Angry, happy faces	33	Masking task	3T	Block, whole-brain and ROI	Emotional vs. neutral	WB: FDR *p* < 0.05; ROI: *p* < 0.001	5
Reker et al., 2010	33f	Sad faces	33	Masking task	3T	Event-related, whole-brain and ROI	Emotional vs. neutral	WB: FDR *p* < 0.05; ROI: *p* < 0.01, SVC	6
Schultheiss et al., 2008	10m, 14f	Sad, angry faces	250	Distraction task (low attentional load)	3T	Block, whole-brain and ROI	Emotional vs. baseline emotional vs. neutral	Uncorrected *p* < 0.005, >10 voxels	13
Simon et al., 2006	8m, 9f	Painful, angry faces	1000	Gender-decision	1.5T	Block and event-related, ROI	Emotional vs. neutral	Uncorrected *p* < 0.005, >4 voxels	16
Sprengelmeyer et al., 1998	2m, 4f	Disgusted, fearful, angry faces	2500	Gender-decision	2T	Block, whole-brain	Emotional vs. neutral	Uncorrected *p* < 0.01, >1 voxel	9
Straube et al., 2004	4m, 6f	Angry faces	1000	Distraction task (low attentional load)	1.5T	Event-related, ROI	Emotional vs. neutral	*p* < 0.005, >50 voxels	3
Suslow et al., 2006	2m, 7f	Angry, fearful, happy faces	33	Masking task	3T	Block, ROI	Emotional vs. neutral	*p* < 0.05	54
Suslow et al., 2009	28m, 23f	Happy, sad faces	33	Masking task	3T	Event-related, whole-brain and ROI	Emotional vs. neutral	WB: FDR *p* < 0.05; ROI: corrected *p* < 0.01, >10 voxels	32
Suslow et al., 2010	30f	Happy faces	33	Masking task	3T	Event-related, ROI	Emotional vs. neutral	*p* < 0.05, SVC	36
Suslow et al., 2013	52m, 58f	Happy, sad faces	33	Masking task	3T	Event-related, whole-brain and ROI	Emotional vs. neutral	WB: *p* < 0.001, >10 voxels; ROI: *p* < 0.005, >10 voxels	4
Vuilleumier et al., 2001	6m, 6f	Fearful faces	250	Dual task (high attentional load)	2T	Event-related, whole-brain and ROI	Emotional vs. neutral	WB: uncorrected *p* < 0.001; ROI: *p* < 0.001, SVC	9
Williams et al., 2004	6m, 6f	Happy, fearful faces	500	Dual task (high attentional load)	3T	Block, ROI	Emotional vs. neutral	*p* < 0.001	3
Williams et al., 2006	7m, 8f	Fearful faces	16.7	Masking task	1.5T	Block, ROI	Emotional vs. neutral	*p* < 0.05, >5 voxel, SVC	8
Yang et al., 2002	6m, 11f	Angry, fearful, happy, sad faces	3000	Gender-decision	3T	Block, ROI	Emotional vs. neutral	*p* < 0.05	5
Yang et al., 2012	14m, 13f	fearful	17	Masking task	3T	Block, whole-brain	Emotional vs. neutral	*p* < 0.001, AlphaSim corrected	13
Total	417m, 545f								624

*m, male; f, female; WB, whole-brain; ROI, region of interest; FDR, false discovery rate; FWE, family-wise error; SVC, small volume correction*.

### Activation likelihood estimation (ALE) meta-analyses

The ALE meta-analysis was carried out in standard MNI space for all selected studies, as well as separate analyses for different experimental paradigms. Foci of contrasts of emotional faces vs. neutral faces were plotted and processed. Eleven of the studies reported coordinates in Talairach space were converted into MNI space by Lancaster's transform (Lancaster et al., 2007). The whole ALE meta-analyses were accomplished by GingerALE 2.3 software (http://brainmap.org/ale/). A subject-based full-width half-maximum (FWHM) (Eickhoff et al., 2009) were applied to the data. The ALE maps were formed by statistical significance corrected for multiple comparisons at the false discovery rate (FDR) *p*-value of 0.01 and cluster extent threshold of 100 mm^3^ according to previous study (Sörös et al., 2009). Sub-analyses for perceptual load, emotional valence, fMRI experimental design were carried out at the same statistical significance threshold (FDR < 0.01, *k* > 100), as well as sub-analyses for studies without using ROI analysis. The comparison between the ALE maps generated by different tasks was obtained by subtraction of ALE values in each voxel using GingerALE too (Eickhoff et al., 2011). A permutation testing with 5000 iterations was made and comparison ALE maps were formed at FDR *p*-value of 0.05, minimum cluster size of 100 mm^3^. All maps of the ALE values were imported into the Mango software (http://ric.uthscsa.edu/mango/index.html) and overlaid onto the “colinbrain” anatomical template normalized to MNI space (Kochunov et al., 2002).

## Results

**Figure 1:**
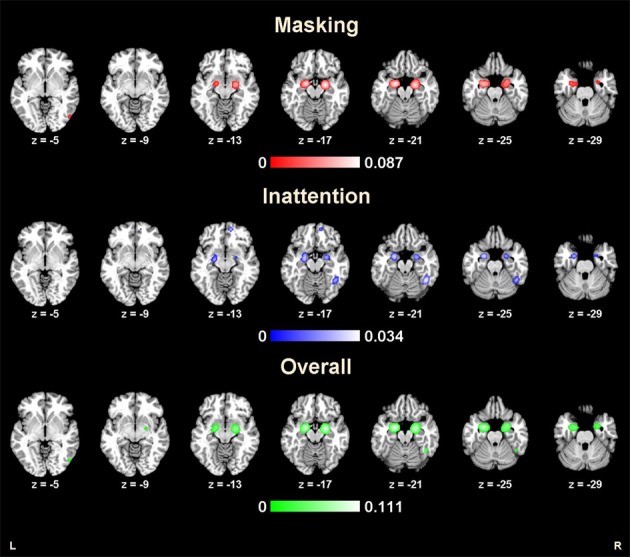
**Results from ALE analysis for masking tasks, inattention tasks, and overall studies (*p* < 0.01; FDR corrected; *k* > 100)**.

Forty-one studies with 830 subjects and 513 foci were identified for inclusion in the ALE meta-analysis (Table 1). Four clusters were identified in the ALE analysis for all 41 studies with 78 emotional vs. neutral contrasts (*p* < FDR 0.01, *k* > 100). As suggested in Figure 1, bilateral amygdala, right middle occipital gyrus (BA 19), and right fusiform gyrus (BA 37) were activated. In 21 studies, 55 foci indicated right amygdala activation; in 20 studies 52 foci indicated left amygdala activation, while 8 foci in one study indicated right middle occipital gyrus activation, 5 foci in 5 studies indicated right fusiform gyrus activation.

### Masking task

The ALE map for masking paradigms (showing 20 studies with 498 subjects and 312 foci) indicated activation in three clusters including bilateral amygdala and right middle occipital gyrus (BA 19) (*p* < FDR 0.01, *k* > 100). See Figure 1. In 10 studies, 37 foci indicated right amygdala activation; 38 foci in 11 studies indicated right amygdala activation, 8 foci in one study indicated right middle occipital gyrus activation.

### Inattention task

The ALE map for inattention paradigms (showing 21 studies with 332 subjects and 201 foci) indicated activation in 5 clusters, including bilateral amygdala, right fusiform gyrus (BA 37), right medial frontal gyrus (BA 10) and left insula (BA 13) (*p* < FDR 0.01, *k* > 100). See Figure 1. 13 foci in 10 studies indicated left amygdala activation; 5 foci in 4 studies indicated right amygdala activation; 7 foci in 7 studies indicated right fusiform gyrus activation; 3 foci in one study indicated right medial frontal gyrus activation; 2 foci in one study indicated left insula activation. All cluster details and ALE values were displayed in Table 2.

**Table 2:** **ALE values of implicit processing of emotional faces**.

	**Side**	**BA**	***X***	***Y***	***Z***	**Volume**	**ALE value**
**OVERALL RESULTS**							
Amygdala	R		22	−6	−18	5128	0.105879
Amygdala	L		−20	−4	−20	4872	0.110621
Middle Occipital Gyrus	R	19	52	−74	−2	264	0.044556
Fusiform Gyrus	R		44	−52	−22	248	0.039371
**MASKING**							
Amygdala	R		22	−6	−18	3616	0.087026
Amygdala	L		−18	−4	−20	3424	0.08703
Middle Occipital Gyrus	R	19	52	−74	−2	264	0.044514
**INATTENTION**							
Amygdala	L		−24	−2	−24	2184	0.030336
Fusiform Gyrus	R		44	−52	−22	1464	0.034016
Amygdala	R		24	−4	−18	1040	0.023138
Medial Frontal Gyrus	R	10	12	54	−14	264	0.02611
Insula	L	13	−42	0	4	208	0.020253

*Results are showed in MNI coordinates. Significance threshold is p < FDR 0.01, k > 100. R, right; L, left*.

Low perceptual load tasks (showing 16 studies with 182 foci) revealed significant activation in bilateral amygdala, right fusiform gyrus (BA 37), right medial frontal gyrus (BA 10) and left insula (BA 13) (*p* < FDR 0.01, *k* > 100). High perceptual load tasks (showing 5 studies with 19 foci) revealed significant activation in right fusiform gyrus (BA 37), right medial frontal gyrus (BA 9), and left parahippocampal gyrus (BA 34) (*p* < FDR 0.01, *k* > 100).

### Sub-analyses for studies using whole-brain analysis

For those studies without using ROI analysis, masking tasks (showing 8 studies with 131 foci) revealed significant activation in bilateral middle occipital gyrus (BA 19) and right lingual gyrus (*p* < FDR 0.01, *k* > 100). Inattention tasks (showing 12 studies with 135 foci) revealed significant activation in right fusiform gyrus (BA 37), left middle occipital gyrus (BA 18), right thalamus, left inferior frontal gyrus (BA 47) and right precuneus gyrus (BA 19) (*p* < FDR 0.01, *k* > 100).

### Sub-analyses for positive and negative emotion

For negative emotional faces, masking tasks (showing 18 studies with 208 foci) revealed significant activation in bilateral amygdala (*p* < FDR 0.01, *k* > 100). Inattention tasks (showing 16 studies with 138 foci) revealed significant activation in bilateral amygdala, right fusiform gyrus, right medial frontal gyrus and left insula (*p* < FDR 0.01, *k* > 100).

For positive emotional faces, masking tasks (showing 9 studies with 88 foci) revealed significant activation in left amygdala (*p* < FDR 0.01, *k* > 100). Inattention tasks (showing 5 studies with 15 foci) revealed no significant activation.

### Sub-analyses for event-related and block design

For event-related studies, masking tasks (showing 8 studies with 87 foci) revealed significant activation in bilateral amygdala, bilateral thalamus, left fusiform gyrus, left inferior frontal gyrus (BA 47), right postcentral gyrus (BA 3), right precuneus gyrus (BA 7), and left middle temporal gyrus (BA 21) (*p* < FDR 0.01, *k* > 100). Inattention tasks (showing 6 studies with 38 foci) revealed significant activation in right fusiform gyrus (BA 37), right thalamus, left parahippocampal gyrus (BA 34) and right medial frontal gyrus (BA 9) (*p* < FDR 0.01, *k* > 100).

For block studies, masking tasks (showing 12 studies with 225 foci) revealed significant activation in bilateral amygdala and right middle occipital gyrus (*p* < FDR 0.01, *k* > 100). Inattention tasks (showing 15 studies with 163 foci) revealed significant activation in bilateral amygdala, right fusiform gyrus (BA 37), right medial frontal gyrus (BA 10), and left insula (BA 13) (*p* < FDR 0.01, *k* > 100).

### Comparison between tasks

**Figure 2:**
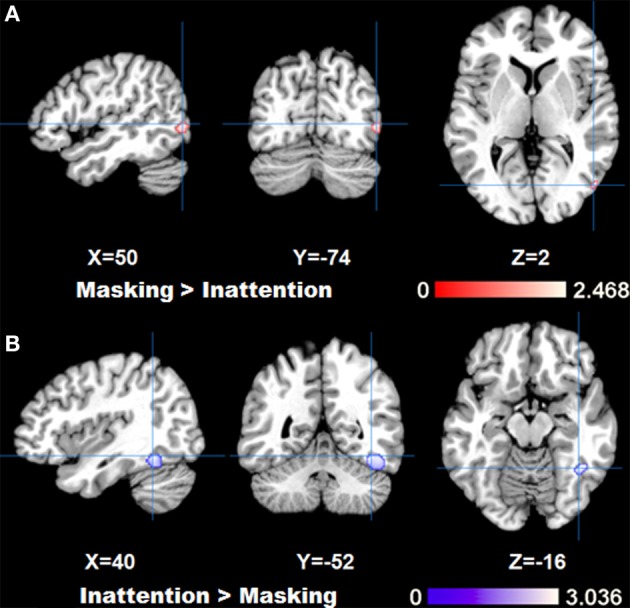
**Results of ALE analysis for comparison between tasks, **(A)** masking>inattention task; **(B)** inattention>masking task showing areas with maximal *z*-values (*p* < 0.05; FDR corrected; *k* > 100)**.

The comparison between two tasks obtained by subtraction revealed significantly higher right fusiform gyrus (BA37) activation for inattention tasks than masking tasks (*p* < FDR 0.05, *k* > 100). Meanwhile, left parahippocampal gyrus, right inferior temporal gyrus and bilateral amygdala were found to be more active in masking tasks than in inattention tasks (*p* < FDR 0.05, *k* > 100). See Figure 2 for maximal activated areas. All cluster details and *Z*-values of the subtracted image were displayed in Table 3.

**Table 3:** **Comparison between tasks**.

	**Side**	**BA**	***X***	***Y***	***Z***	**Volume**	***Z***
**INATTENTION>MASKING**							
Fusiform Gyrus	R	37	40	−52	−16	1168	3.035672
	R	37	45	−50	−16		2.947843
**MASKING>INATTENION**							
Parahippocampal Gyrus	L	28	−12	−4	−16	336	2.180776
	L	34	−12	0	−20		2.149434
Inferior Temporal Gyrus	R		50	−74	2	240	2.467659
Amygdala	L		−34	−2	−24	168	2.006527
Amygdala	R		26	4	−20	112	1.920459

*Results are showed in MNI coordinates. Significance threshold is p < FDR 0.05, k > 100. R, right; L, left*.

## Discussion

The present study used ALE meta-analysis to explore brain activation in response to emotional faces by two primary implicit emotional processing tasks and paradigms. Through this method it was possible to combine studies, perform statistical analyses of the whole brain, and generate activation maps based on coordinates. To our knowledge, this is the first study to compare the mechanisms underlying the two tasks of implicit emotional processing. The two tasks of implicit processing induced activation of distinct areas, with masking tasks preferentially associated with inferior temporal gyrus and limbic areas such as parahippocampal gyrus and amygdala, while inattention tasks preferentially associated with right fusiform gyrus. Implicit emotional faces activated brain regions such as bilateral amygdala, right middle occipital gyrus and right fusiform gyrus across both tasks.

### Implicit processing of emotional faces

Many studies have confirmed that the amygdala plays an important role in the implicit emotional face processing (Morris et al., 1996; Whalen et al., 1998; Williams et al., 2004). Whalen et al. (1998) regarded the amygdala as a vigilance system, functioning in conjunction with the cognition of ambiguous stimuli with biological relevance, such as emotional faces. Our overall results showed greatest activation in bilateral amygdala, which is consistent with previous studies (Baas et al., 2004; Brooks et al., 2012). According to LeDoux (1996), there exists a rapid neural pathway for salient emotional stimuli from amygdala to visual cortex. Fusiform gyrus is also regarded to be involved in this rapid neural pathway. A lot of studies have confirmed that fusiform gyrus is associated with emotional face identification and perception (Morris et al., 1999; Critchley et al., 2000a; Kanwisher and Yovel, 2006; Said et al., 2011). Research also showed fusiform gyrus activation for highly salient stimuli even without conscious perception (Litt et al., 2011), which is supported by our results. Middle occipital gyrus is identified as an important part of visual cortex, thus it is not surprised to find it activated in our results for emotional face processing (Lindquist et al., 2012).

### Comparison between two tasks

Adolphs (2002) has proposed a classification of three stages in emotional processing: first there is a rapid early processing for highly salient stimuli; then detailed processing occurs and an emotional response is aroused; third stage is when emotional recognition finally takes place. Evidence from ERP studies has revealed the time courses of these processes. Dolan (2002) found preconscious processing of emotional stimuli occurs at 100–120 ms after stimulus presentation, Liddell et al. (2004) found the N2 ERP component at 200–300 ms post-stimulus range, represents an automatic, attentional response. According to Tamietto and de Gelder (2010), masking task occurs during the early stage of implicit emotional processing, while inattention task occurs during later stage of processing, especially when attention is limited (Wolbers et al., 2006). In our results, masking tasks showed only amygdala and visual area activation, while inattention tasks showed a complex network, with great activation in the fusiform gyrus, medial frontal gyrus and insula. A review by Vuilleumier and Pourtois (2007) suggested that distributed brain areas like amygdala, insula, ventral prefrontal cortex and superior temporal cortex might be involved in distinct latencies of emotional processing. Medial frontal gyrus is indicated to participate in the conscious experience of emotion (Fusar-Poli et al., 2009b). Insula is suggested to play a role in identifying the emotional significance of stimuli (Phillips et al., 2003). Paralimbic regions such as insula play an important role in conveying information between subcortical structures such as amygdala and other cortical structures (Lindquist et al., 2012). These evidence may support that inattention tasks reflect both early and later stages of emotional processing (Phelps et al., 2001). Researchers found that a subcortical pathway to the amygdala-hippocampal area for implicit emotional processing works in parallel with a cortical route to the prefrontal cortex, which is necessary for conscious identification (LeDoux, 1996; Morris et al., 1999; Phillips et al., 2003). The subcortical pathway responds rapidly to the stimuli, while the cortical pathway evaluates and regulates the response. Our results suggested that masking task preferentially activated limbic areas such as parahippocampal gyrus and amygdala, as well as inferior temporal gyrus, which is a part of visual areas, and has strong connections to the amygdala (Pessoa, 2008). Inattention task preferentially activated the fusiform gyrus, which has been stated above to be associated with emotional identification and perception. Therefore, these evidence may support that masking task reveals the early stage of emotional processing, while inattention task reveal later stage of pre-attentive processing which may serve as a transitional stage from implicit processing toward explicit processing (Phan et al., 2004).

As a priori regions of interest might be a confounding factor, we also ran a separate analysis excluding the studies using only ROI analysis. For masking tasks, studies using whole-brain analysis showed activation in visual areas such as middle occipital gyrus and lingual gyrus, which have been suggested to be involved in early processing of faces (Adolphs, 2002; Fusar-Poli et al., 2009b). For inattention tasks, activation in fusiform gyrus, middle occipital gyrus, thalamus, inferior frontal gyrus and precuneus gyrus was found. Thalamus contributes to the generation of emotional responses (Lane, 2008). Inferior frontal gyrus is known to be involved in the implicit processing of emotional faces (Adolphs, 2002; Phillips et al., 2003, 2004; Killgore and Yurgelun-Todd, 2004), and also serves as a part of core regions of ventral frontoparietal network (Corbetta et al., 2008). Precuneus gyrus is a part of superior parietal lobule, which is one of the core regions of dorsal frontoparietal network (Corbetta et al., 2008). According to Corbetta et al. (2008), ventral network is involved in directing attention to salient stimuli, and dorsal network is involved in goal-directed attentional selection. The two networks interact with each other to reorient stimulus-driven and top–down attention. Thus, this evidence also supports that inattention task reveal later stage of emotional processing, where pre-attentive processing takes place.

The current study emphasized the importance of experimental tasks selection in the exploration of different domains. For example, inattention tasks may be useful for studies on emotional processing influenced by attentional processes, such as attentional bias, attention deficit, and anxiety disorders (Critchley et al., 2000b; Straube et al., 2004; Anderson et al., 2007; Palm et al., 2011). The sensitivity of the masking tasks in implicit processing may make it especially useful in the detection of vulnerabilities to mental illness such as major depression disorder and subsequent primary prevention (Etkin et al., 2004; Rauch et al., 2007).

### Sub-analyses for emotional valence, perceptual load, fMRI designs and contrasts

Sub-analyses for negative emotional faces revealed almost identical results to the main results of both masking and inattention tasks. Sub-analyses for positive emotional faces revealed amygdala activation for masking tasks. However, there were not enough foci (only 15) for inattention tasks to reveal significant results. Different emotional valence was indicated to have different activation patterns (Fusar-Poli et al., 2009b; Vytal and Hamann, 2010). However, this study focused on task difference rather than emotional valence difference. Moreover, the included studies with negative emotional faces contributed to the main results for the most part. Similarly, there were not enough foci (only 19) for high perceptual load tasks to reveal reliable results. Low perceptual load tasks contributed to the main results for the most part. Future studies should include enough studies for different emotional categories and cognitive demands to evaluate the effects of emotional valence and perceptual load. Sub-analyses for block studies revealed almost identical results to the main results too. Sub-analyses for event-related studies showed variant activations other than the main results, including the thalamus, fusiform gyrus, inferior frontal gyrus, postcentral gyrus, precuneus gyrus, and middle temporal gyrus for masking tasks; thalamus and parahippocampal gyrus for inattention tasks. These regions were all suggested to be involved in emotional face processing (Brooks et al., 2012; Fusar-Poli et al., 2009b). However, this implied that heterogeneity in fMRI designs would cause bias in the meta-analysis results.

### Limitations

The present study used the ALE meta-analysis method which is more reliable than a single study. The expanded sample size increased statistical power. In addition, the entire scope of activation was provided. However, there were some limitations to this method of study. First, meta-analysis inherently lacks data homogeneity, thus making conclusions open to further study. Second, The ALE meta-analysis method does not account for strength of activation. It is therefore possible that important brain regions with lower activation levels may be overlooked. Third, the conversion between different coordinate systems and heterogeneous definitions of anatomical labels may also affect results.

Although masking tasks are widely accepted and used to explore implicit processing, there is no good evidence to suggest that masked faces are processed completely subliminally. Pessoa (2005) reported that more than 60% of the subjects in their study reported actually seeing the masked stimuli, indicating individual differences in sensitivity to emotional faces. Although in the current study, most of the articles using a masking task that were included in the meta-analysis provided a probe test, demonstrating that subjects were not aware of the masking stimulus. As to the inattention tasks, Phan et al. (2004) believed that tasks involving cognitive effort do not always distract attention from emotional stimuli. In fact, the fluctuation of attention could hinder the strength of comparisons made between distractors (emotional stimuli) and targets (non-emotional stimuli).

## Conclusion

Neuroimaging studies of the implicit emotional face processing were analyzed and compared using the ALE meta-analysis method. There were distinct and overlapping results between masking tasks and inattention tasks. Masking tasks implied early stages of implicit emotional processing while inattention tasks suggested later stages of implicit emotional processing. This meta-analysis provides a new point of view to evaluate the effects of different tasks and emphasizes the importance of experimental task selection in the exploration of different domains.

### Conflict of interest statement

The authors declare that the research was conducted in the absence of any commercial or financial relationships that could be construed as a potential conflict of interest.
